# NMO-IgG and AQP4 Peptide Can Induce Aggravation of EAMG and Immune-Mediated Muscle Weakness

**DOI:** 10.1155/2018/5389282

**Published:** 2018-05-22

**Authors:** Tehila Mizrachi, Livnat Brill, Malcolm Rabie, Yoram Nevo, Yakov Fellig, Mayan Zur, Dimitrios Karussis, Oded Abramsky, Talma Brenner, Adi Vaknin-Dembinsky

**Affiliations:** ^1^Department of Neurology and The Multiple Sclerosis Center, The Agnes-Ginges Center for Neurogenetics, Hebrew University, Hadassah Medical Center, Ein Karem, Jerusalem 91120, Israel; ^2^Schneider Children's Medical Center of Israel, Tel Aviv University, Tel Aviv, Israel

## Abstract

Neuromyelitis optica (NMO) and myasthenia gravis (MG) are autoimmune diseases mediated by autoantibodies against either aquaporin 4 (AQP4) or acetylcholine receptor (AChR), respectively. Recently, we and others have reported an increased prevalence of NMO in patients with MG. To verify whether coexisting autoimmune disease may exacerbate experimental autoimmune MG, we tested whether active immunization with AQP4 peptides or passive transfer of NMO-Ig can affect the severity of EAMG. Injection of either AQP4 peptide or NMO-Ig to EAMG or to naive mice caused increased fatigability and aggravation of EAMG symptoms as expressed by augmented muscle weakness (but not paralysis), decremental response to repetitive nerve stimulation, increased neuromuscular jitter, and aberration of immune responses. Thus, our study shows increased disease severity in EAMG mice following immunization with the NMO autoantigen AQP4 or by NMO-Ig, mediated by augmented inflammatory response. This can explain exacerbation or increased susceptibility of patients with one autoimmune disease to develop additional autoimmune syndrome.

## 1. Introduction

Neuromyelitis optica (NMO), also known as Devic's disease, is a central nervous system (CNS) autoimmune disease that preferentially affects the spinal cord and optic nerve [1]. The disease is mediated by autoantibodies against aquaporin 4 (AQP4) [2]. These antibodies have been proven pathogenic in NMO by several methods including complement-dependent cytotoxicity, antibody-dependent cell-mediated cytotoxicity, and induction of inflammation with a prominent granulocyte and macrophage response, which lead to secondary oligodendrocyte injury, demyelination, and neuronal injury [3].

Myasthenia gravis (MG) is a well-recognized antibody-mediated disease affecting the neuromuscular junction, caused by immunoglobulin G (IgG)1- and IgG3-complement, activating antibodies against the nicotinic acetylcholine receptor (AChR, AChR-Ab) in around 85% of patients [4].

Both AQP4-Ab-positive NMO and AChR-Ab-positive MG are associated with other organ-specific and systemic autoimmune diseases [5–7]. Despite the rarity of MG and of NMO, in recent years, there is considerable evidence for increased susceptibility of NMO in patients with MG. Our and others' studies have linked NMO to patients previously diagnosed with MG and pointed common immunological abnormalities between the two diseases [8–15].

Although AQP4 is expressed also outside the CNS, in muscles, lungs, and kidneys, until recently, no disease was described in those organs. There are currently indications that there might be mild muscle pathology in patients with NMO [16–18].

Currently, there is no satisfactory animal model of NMO. In order to study the pathogenesis of NMO and to test candidate therapies, it is important to have an animal model of the disease [19]. Several animal studies have shown that AQP4 antibodies are not pathogenic via simple transfer of AQP4 antibodies into the circulation of naive animals. In order to cause NMO pathology, NMO-IgG should reach the CNS parenchyma by penetrating through the blood-brain barrier (BBB). This was established using preexisting CNS inflammation in the experimental autoimmune encephalitis (EAE) model, to cross the BBB, or via direct intracerebral injection of recombinant NMO-IgG [20–23]. Direct administration of NMO-IgG into the CNS tissue, without coinjection of complement, produced NMO-like lesions with astrocyte and AQP4 loss [24]. By injection of NMO-IgG into mice lacking complement inhibitor, Zhang et al. induced long extensive myelitis comparable to the myelitis in humans with NMO [25]. Recently, several studies showed that induction of NMO-like syndrome can be induced by the transfer of AQP4-reactive T-cells directed to the second extracellular loop of AQP4. These T-cells were derived from AQP4 null mice and injected to wild type or to B cell-deficient mice [23, 26, 27].

The present study was aimed at establishing an animal model for NMO together with MG, based on previous observation of increased NMO susceptibility in patients with MG. We used experimental autoimmune MG (EAMG) mice immunized with Torpedo AChR and subjected the animals to passive transfer of NMO-IgG or to immunization with AQP4-derived peptide for inducing NMO and MG models.

Our study shows that injection of either AQP4 peptide or NMO-Ig to naive mice caused increased fatigability and that the same agents' administered to EAMG mice significantly increased disease severity mediated by muscle weakness.

## 2. Materials and Methods

### 2.1. EAMG and NMO Induction and Clinical Evaluation

Induction of EAMG C57BL/6JOlaHsd mice were purchased from Harlan Laboratories (Rehovot, Israel) and were housed under specific pathogen-free conditions in the animal facility of the Hebrew University Medical School, in accordance with NIH guidelines for the care and use of laboratory animals. Torpedo AChR was purified from *Torpedo californica* as previously described [28].

Purified Torpedo AChR (25 *μ*g and 5 mg/ml of M. tuberculosis H37Ra, (Difco, Detroit MI)), emulsified in complete Freund's adjuvant (CFA), was subcutaneously (s.c.) injected into the hind footpads and into the flank of 6-7-week-old C57BL female mice as previously described [29]. The clinical status was graded as the following: (0) no weakness or fatigue; (1) mildly decreased activity, weak grip with fatigue, weight loss > 3% body weight in one week; (2) moderate weakness accompanied by weak grip, 5–10% weight loss; (3) moderate-severe weakness, hunched back posture at rest, head down, and forelimb digit flexed, tremulous ambulation, 10% weight loss; and (4) severe general weakness, weak grip, weight loss > 10% [29].

#### 2.1.1. Preparation of NMO-Ig

Plasma exchange fluid was collected during the procedure from clinically definite, seropositive NMO patient according to the guidelines and with the approval of the Hebrew University's Bioethics Committee. The diagnosis of NMO was based on positive titer of anti-AQP4 antibodies and typical clinical symptoms, including optic neuritis and myelitis [30]. Immunoglobulin (Ig) was purified by saturated ammonium sulfate precipitation followed by dialysis.

#### 2.1.2. Injection of NMO-Ig or AQP4 Peptide

Seven groups of mice were used in 3 different sets of experiments:

1. Twenty-one control EAMG
2. Thirteen naive mice injected intraperitoneally (i.p.) with Ig isolated from NMO patient, 4 weeks after the induction of EAMG (the amount of Ig was 17.5 mg protein; Ig injection was repeated 3 times with 10-day intervals between each injection)
3. Six EAMG mice immunized with 40 *μ*g AQP4 peptide p249-323 (Alomone Labs, Israel), AQP4 peptide emulsified in complete Freund's adjuvant (CFA)
4. Thirteen naive mice that were injected i.p. with 17.5 mg Ig protein (Ig injection was repeated 3 times with 10-day intervals between each injection)
5. Fifteen naive mice immunized with 40 *μ*g AQP4 peptide p249-323 (Alomone Labs, Israel), AQP4 peptide emulsified with CFA
6. Six naive mice injected with CFA alone
7. Eight naive untreated mice

All the animals were weighed and inspected once a week during the first month and daily after AQP4 peptide and NMO-Ig injection to evaluate muscle weakness.

In addition, 10 naive and EAMG mice were injected with scrambled AQP4 peptides (amino acids 207-221, 217-231, 222-239, and 299-213, chosen from loop E- and C-terminus regions) in CFA and inspected as above. Those animals did not develop any measured clinical weakness (data not shown).

#### 2.1.3. Anti-AChR Ab Determination

Sera from EAMG animals were assayed by direct radioimmunoassay for anti-T-AChR, or mouse M-AChR. All the EAMG mice displayed high anti-T-AChR and/or anti-mouse AChR levels, with the serum mean ± SE values of 82.1 ± 16.0 nM for anti-T-AChR Abs and 19.9 ± 1.8 nM for anti-M-AChR.

#### 2.1.4. Repetitive Nerve Conduction Studies (RNS)

Mice were anesthetized by i.p. injection of 157 mg/kg ketamine + 3.5 mg/kg xylazine and immobilized on silver foil over an AccuBlock™ at 41°C to maintain rectal temperature between 36-37°C. The left hind limb was abducted slightly and immobilized with the knee and ankle in slight flexion and shaved. An electromyography (EMG) machine was used to assess motor nerve conduction, RNS, and single fiber electromyography (SFEMG). Baseline motor conduction filter settings were 0.01–10 kHz, amplifier gain 2 mV/division, sweep duration 2 ms/division, and stimulus duration 0.1 ms (rectangular pulses). Disposable sensory needle electrodes (SNEs) (28G, Alpine Biomed ApS) were used for both stimulating and recording motor conduction and RNS studies. The left sciatic-tibial nerve was stimulated as described by Osuchowski et al. [31]. The stimulator anode SNE was advanced subdermally lateral and parallel to the spine 11 mm from the tail-base, and cathode SNE was inserted subdermally 3 mm lateral and parallel to the anode. For recording, an active pick-up SNE was inserted into the gastrocnemius muscle and reference SNE was placed subdermally over the Achilles tendon. Baseline supramaximal sciatic-tibial nerve compound muscle action potential (CMAP) amplitudes were recorded from gastrocnemius, followed by a 5 Hz train of RNS at supramaximal intensity. A decrement was calculated as percent amplitude drops between the first and fifth CMAPs evoked by a train of 10 impulses, which were determined in two sets of repetitive stimulation for each animal. A reduction of 10% or more indicated neuromuscular transmission dysfunction [32]. Normal muscle showed no decrement in action potential amplitude.

#### 2.1.5. Concentric Needle Stimulation SFEMG

Anesthetized mice were kept immobilized, and temperatures maintained as above. Stimulation SFEMG was performed by direct extramuscular nerve stimulation and was picked up by concentric electromyography needle (CN). SFEMG filter settings were 0.5–10 kHz, amplifier gain 1 mV/division, and sweep duration 1 ms/division. Left sciatic-tibial nerve stimulation [31] was initially of low rate and intensity (3 Hz, 0.1–0.3 mA), stimulus duration 0.1 ms (rectangular pulses) to obtain correct CN positioning and spike wave forms. A disposable CN electrode (30G, Alpine Biomed ApS) was inserted into the midpoint of the twitching gastrocnemius, and reference (anode) via a surface cup electrode (Alpine Biomed ApS, 10 mm diameter, gold-plated) was affixed with gel onto the skin 1.5 cm from the CN insertion site. The motor unit potential recorded with a CN electrode represents the sum of single fiber action potentials (SFAPs) from all muscle fibers from a single motor unit that lays within the uptake range of the electrode [33]. The “apparent single fiber action potentials” (ASFAPs) spikes were identified with amplitudes > 200 IV, rise time < 200 ìs, [33]. Thereafter, stimulation intensity was increased to abolish false positive blocking due to insufficient stimulus strength, to 10 Hz, with muscle movement minimized. Five to fourteen apparent single muscle fiber potentials per animal were used for the analysis. The jitter of each ASFAP was computed as the mean consecutive difference (MCD) of 100 consecutive intervals between stimulus artifact and muscle fiber potential. A jitter (MCD) value ≤ 5 was rejected as being due to muscle fiber splitting or technical factors [32, 34]. Neuromuscular blocking was calculated as the percent of all fibers in which any blocking was seen among the 5–14 ASFAPs studied per animal.

#### 2.1.6. Grip Strength Analysis

Starting from week 10 and for 8 consecutive weeks, forelimbs' muscle strength was determined using the electronic grip strength meter (Columbus Instruments). Forelimb muscle strength was assessed once a week according Tanase et al. [35]. Five measurements were performed on the animal's forelimbs. The three highest measurements in each mouse group were averaged to give the mean strength score. The results are given as grams force divided by body weight (gr).

#### 2.1.7. Cell Culture and Cytokine and Chemokine Production

Pooled lymphocytes from spleens were harvested from naive mice or from mice immunized 7 weeks earlier with T-AChR in CFA or with NMO-Ig or AQP4 peptide. 1 × 10^6^ cells were cultured in 24-well plates, in a total volume of 0.5 ml. stimulated with purified anti-CD3 antibodies and incubated for 24 h to 96 h. The conditioned media were collected, and the following cytokines interferon *γ* (IFN*γ*), interleukin 6 (IL-6), and interleukin 10 (IL-10) were tested using enzyme-linked immunosorbent assay (ELISA) kits (Biolegend) according to the manufacturer's instructions, as previously reported [36].

#### 2.1.8. RNA Purification and cDNA Synthesis

Total RNA was prepared using a 5 Prime RNA kit (GmBh Hamburg, Germany). cDNA was prepared from 1 mg of total RNA, using a qScript cDNA synthesis kit (Qunta).

#### 2.1.9. Cytokines and Chemokines Expressed in Muscles of EAMG- and NMO-Ig- and AQP4 Peptide-Injected Animals

The following cytokines and chemokines were tested on muscle specimens using RT-PCR. The reaction mixture contained 40 ng cDNA and 4 *μ*l buffer containing 300 nM of the appropriate forward and reverse primers and PerfeCTa SYBR Green FastMix ROX (Quanta) in a total volume of 7 *μ*l. Gene amplification was carried out using the GeneAmp 7000 Sequence Detection System (Applied Biosystems). The results are expressed as relative quantification (RQ), and the fold increase of gene expression in samples from concanavalin A (Con A) stimulated cells above those from control cells. The results for gene expression were normalized to the HPRT gene; the expression of which was not changed under stimulation conditions. The primers used were HPRT: fwd: 5′TCCTCCTCAGACCGCTTTT3′ and rev: 5′CCTGGTTCATCATCGCTAATC3′; TGF: fwd: 5′TCAGACATTCGGGAAGCAGT3′ and rev: 5′ACGCCAGGAATTGTTGCTAT3′; IP-10: fwd: 5′GGATGGCTGTCCTAGCTCTG3′ and rev: 5′ATAACCCCTTGGGAAGATGG3′; CXCR3: fwd: 5′TGGGGCGGTGGCTGCTGTGCT3′ and rev: 5′TGCAGAGGCCAGGGCCGAAAACCC3′; atrogin: fwd: 5′AAGAAGAGAGCAGTATGGGGTCA3′ and rev: 5′ACGGATGGTCAGTGCCCTT3′; MCP-1: fwd: 5′ACCTGTAAATGCCATGCAAGT3′ and rev: 5′TGTCTTCCATTTCCTTTGATTT3′; and IL-6: fwd: 5′AGTTGCCTTCTTGGGACTGA3′ and rev: 5′TCCACGATTTCCCAGAGAAC3′.

#### 2.1.10. Pathology

On the day of sacrifice, the optic nerve, spinal cords, and brain were harvested and fixed in 4% paraformaldehyde, dehydrated, and embedded in paraffin. Longitudinal sections were cut to include the majority of the length of the spinal cord, containing both gray and white matter, and stained with hematoxylin-eosin.

#### 2.1.11. Statistical Analysis

The data were analyzed using Student's *t*-test and one-way ANOVA. *P* < 0.05 was considered statistically significant.

## 3. Results

### 3.1. NMO-Ig and AQP4 Peptide Aggravate EAMG and Induce Immune-Mediated Muscle Weakness

Noting that NMO clinical symptoms and the presence of the pathogenic anti-AQP4 antibodies have recently been reported in patients with MG [9, 12] and that anti-AChR antibodies were found in 11% of NMO patients [10], we examined whether and how one disease affects the severity of the autoimmune course of the other disease. EAMG mice were injected with AQP4 antibodies or peptide, and we verified the disease pattern and determined the effect of AQP4 antibodies or peptide on the immune pathophysiology in the CNS and in the muscle.

**Figure 1:**
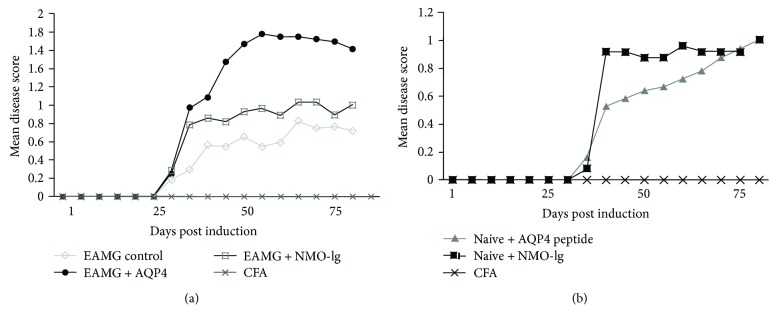
Aggravation of muscle weakness symptoms in naive and EAMG mice by exposure to NMO-Ig or AQP4 peptide. (a) Injection of AQP4 peptide or NMO-IgG in addition to EAMG induction resulted in aggravation of EAMG weakness symptoms. (b) Mice injected with AQP4 peptide or NMO-IgG had significant muscle weakness as compared to CFA-injected mice. Results are the mean of 3 separated experiments. EAMG control *n* = 16, EAMG + AQP4 peptide *n* = 6, EAMG + NMO-Ig *n* = 7, AQP4 peptide *n* = 9, NMO-Ig *n* = 6, and CFA *n* = 6.

A total of 82 mice (21 with EAMG alone, 13 with EAMG + NMO-Ig, 6 with EAMG + AQP4 peptide, 13 injected with NMO-Ig alone, 15 injected with AQP4 peptide alone, 6 with CFA, and 8 naive mice) were included in this study in 3 different sets of experiments. EAMG mice that received AQP4 antibodies or peptide developed increased EAMG scale severity of 0.82 ± 0.01 and 1.5 ± 0.02, respectively, with increased muscle weakness and fatigability, compared with 0.62 + 0.01 in mice with EAMG alone (Figure 1(a) and Table 1). Thus, the additional immunological challenge to AQP4 components significantly aggravated EAMG. Moreover, exposure to AQP4 antibodies or peptide alone also induced measurable impairment in muscle force. Naive mice that were subjected to AQP4 antibodies or AQP4 peptide developed mild fatigability that was quantified as 0.72 ± 0.01 and 0.63 ± 0.02, respectively, in disease scores (Figure 1(b)). The naive mice injected with NMO-Ig or AQP4 peptide displayed weakness, which was expressed by mildly decreased activity (relative to CFA, scrambled peptides, and Ig-injected control mice).

Aside from the clinical severity score, several measurements of muscle strength were performed, including weight changes and grip strength, and a fraction of the mice were subjected to RNS and SFEMG.

### 3.2. Forelimb Grip Strength Test

**Figure 2:**
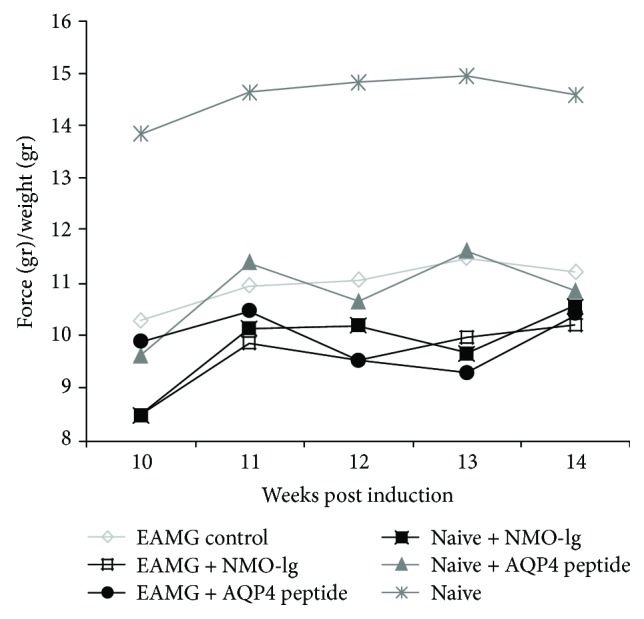
Forelimb muscle strength in AQP4 peptide- or NMO-IgG-injected mice. Using an electronic grip strength meter, the forelimb force was determined once a week. All the groups showed a significant reduction in forelimb strength in comparison to the CFA control mice. Results are the mean of 2 separated experiments with 4 to 7 mice in each group.

In order to quantify muscle weakness symptoms in the different mice groups, we performed a longitudinal assessment with 8 weekly measurements of the forelimb grip strength test. Grip strength was determined and divided by the mouse weight. As shown in Figure 2, and Table 1, all 5 experimental groups EAMG, NMO-Ig, AQP4 peptide, EAMG plus NMO-Ig, and EAMG plus AQP4 peptide (except the control CFA) showed significant reduction in the force values of 21.1–30.4% and increased fatigability compared to CFA (*P* < 0.05).

### 3.3. Measurements of CNS and Optic Nerve Histopathology

Since NMO disease in humans affects mainly the spinal cord (SC) and the optic nerves (ON), we studied the brain, SC, and ON obtained from 3 mice in each group (control naive mice, EAMG alone, and EAMG injected with NMO-Ig or with AQP4 peptide). To our surprise, although there was increased disease severity in mice with EAMG injected with either AQP4 peptide or NMO-Ig, no CNS pathological abnormalities were observed in these mice (data not shown).

### 3.4. Evidence of Neuromuscular Junction Pathology on Repetitive Nerve Stimulation and Single Fiber Electromyography

**Figure 3:**
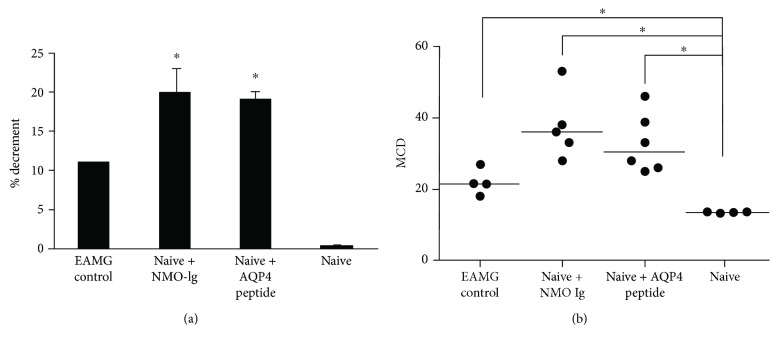
The injection of AQP4 peptide or NMO-Ig induces pathological repetitive nerve stimulation and jitter. (a) Changes in the 5th/1st muscle action potential amplitude ratio calculated as percent decrement and expressed as the mean ± SE in EAMG mice and NMO-Ig- and AQP4 peptide-injected mice. Naive mice had no decrement. (b) NMO-Ig or AQP4 peptide increased neuromuscular mean consecutive difference (MCD). The baseline MCD value of the control CFA-injected mice was 13.5 ± 0.1 *μ*s. The MCD values of EAMG mice as well as mice injected with AQP4 peptide or NMO antibodies were significantly elevated and pathologic. ^∗^*P* < 0.05.

Since there was no evidence of CNS pathology, to verify the possibility that the fatigability of mice injected with AQP4 peptide or NMO-Ig originated from neuromuscular junction pathology using a nonbiased test, the mice were tested for their response to RNS and SFEMG. Compound muscle action potentials (CMAPs) from the gastrocnemius muscle were recorded in naive mice injected with NMO-Ig or AQP4 peptide alone and compared to EAMG- and CFA-injected mice. EAMG mice displayed a decrement in CMAP amplitudes. The baseline pathological decrement ranged from 11 ± 0.66% to 20 ± 3%, as shown in Figure 3(a) (with decrement above 10% considered abnormal).

Concentric needle stimulation SFEMG was performed on the same mice groups described above; the mean jitter values (mean MCD) of CFA-injected mice, EAMG mice, and mice injected with NMO-Ig or AQP4 peptide alone were calculated. The CFA-injected mice showed a baseline jitter (mean MCD) of 13.5 ± 0.1 ìs. EAMG control and mice injected with NMO-Ig or AQP4 peptide showed significantly increased neuromuscular jitter as shown in Figure 3(b) (22.03 ± 1.8 ìs, 35.9 ± 2.2 ìs, and 32.2 ± 6.5 ìs, resp., *P* < 0.01). We also observed neuromuscular blocking in 50% of the EAMG controls, in 100% of the mice injected with NMO-Ig, in 50% of mice injected with AQP4 peptide, and none in the naive CFA-injected mice. All of these electrophysiological findings support neuromuscular junction pathology.

### 3.5. Immune Responses in AQP4 Peptide or NMO-Ig Injected to Both Naive and EAMG Mice

To investigate whether the systemic immune response differs in EAMG and EAMG/NMO mice, we studied the spleen and lymph node cell repertoire using flow cytometry and the secretion of cytokines from spleen cells stimulated by anti-CD3 using ELISA assay.

**Figure 4:**
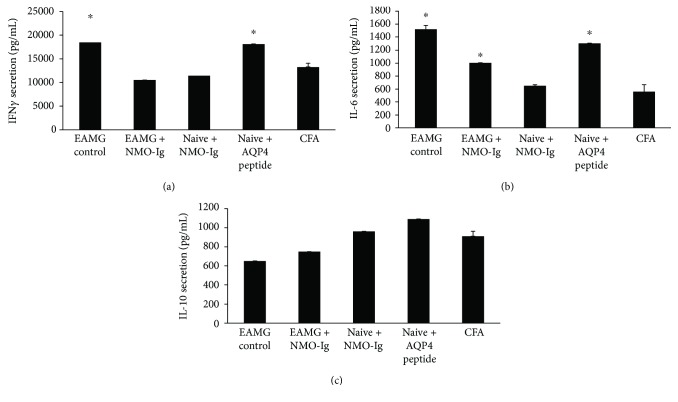
Increased proinflammatory cytokine secretion. Splenocytes derived from EAMG control, EAMG with NMO-Ig, and from naive mice injected with NMO-Ig or AQP4 peptide, or CFA, was activated by anti-CD3. Cell-free supernatants were tested for the cytokine content by specific ELISA. IFN*γ* was tested after 24 h of stimulation, IL-6 (48 h of stimulation), and IL-10 (after 72 h). Results are the mean of 2 different sets of experiments of 3–5 mice in each group and are expressed as the mean ± SE. ^∗^*P* < 0.05 compared to CFA-injected mice.

Lymphocyte cultures derived from CFA-injected mice were compared with lymphocytes from EAMG injected with AQP4 peptide or with NMO-Ig and cultures derived from naive mice that were injected with this peptide or antibodies. As shown in Figure 4(a), we found significantly increased IFN*γ* secretion when the cells were stimulated with anti-CD3, in both EAMG control mice and naive mice injected with AQP4 peptide (18,469 ± 830, 18,086 ± 1121, resp., and 13,230 ± 848 in the CFA injected mice, *P* < 0.05). Significantly increased IL-6 secretion (compared to CFA-injected mice) was detected (Figure 4(b)), in EAMG control mice, in EAMG mice injected with NMO-Ig, and in naive mice injected with AQP4 peptide (1521 ± 59, 1004 ± 17.4 1303.7 ± 54, resp., and 560 ± 106.5 in the CFA-injected mice, *P* < 0.05). IL-10 secretion did not change significantly (Figure 4(c)).

Flow cytometry analysis of lymphocytes derived from the various mouse groups measuring CD4+, CD8+, CD19+,CD19+/CD27+, CD11b+,CD11c, and MHC-class II cells did not show any statistical difference between the treated groups as compared with the CFA-injected group. Although, EAMG + NMO mice had a significantly more severe disease score and aggravated impairment in force compared with the EAMG mice alone. No significant differences were found in these immune cell markers in these groups (data not shown).

### 3.6. Expression of Inflammatory Markers in Muscle

**Figure 5:**
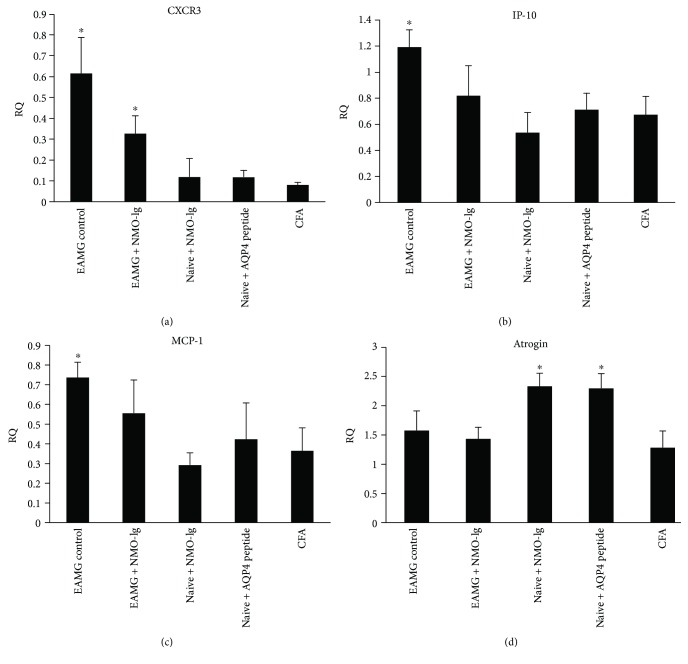
Changes in the expression of muscle markers. The expression of proinflammatory chemokines CXCR3 (a), IP-10 (b), and MCP-1(c) as well as the atrophy marker atrogin (d) were measured by RT-PCR. Results are the mean of 3 different sets of experiments of 3–5 mice in each group and are expressed as the mean ± SE. ^∗^*P* < 0.05.

We were interested to verify whether the increased severity of muscle fatigability in the EAMG + NMO mice was mediated by the expression of muscle proinflammatory markers. Using RT-PCR, the expression levels of cytokines (IL-6, transforming growth factor *β* (TGF *β*)), chemokines, and chemokine receptors C-X-C motif chemokine receptor 3 (CXCR3), interferon gamma inducible protein 10 (IP-10), and monocyte chemoattractant protein 1 (MCP-1), and the atrophy-associated gene atrogin-1 were analyzed. As shown in Figure 5, our study confirmed the findings of increased MCP-1, CXCR3, and IP-10 in EAMG mice as compared to CFA control mice (*P* = 0.05, and 0.06, resp.) [37]. Interestingly, expression of the atrogin-1 muscle atrophy-associated gene was increased in mice that received NMO-Ig and in those injected with AQP4 peptide, while atrogin-1 expression did not change in the EAMG mice (Figure 5(d)). Proinflammatory markers (MCP-1, CXCR3, and IP-10, Figure 5(a)–5(c)) were increased in EAMG mice, while expression of IL-6 and TGF*β* did not change (data not shown).

## 4. Discussion

In the present study, we observed that coexisting autoimmune disease—EAMG—can be exacerbated by the NMO antigen or antibody. The injection of AQP4 peptide or NMO-Ig increased disease severity in these mice and induced mild fatigability in naive mice.

Muscle weakness was expressed by decreased grip force in AQP4 peptide- or NMO-Ig naive-injected mice. This weakness could be attributed to CNS and/or neuromuscular inflammation. However, we did not find evidence for CNS inflammation by brain, spinal cord, and optic nerve histopathology, arguing against major CNS involvement in these mice. On the other hand, we found clear neuromuscular pathology including abnormal neuromuscular transmission with decrement response on RNS and prolonged jitter and blocking. The neuromuscular pathology can be either inflammatory or degenerative. The abnormal neuromuscular transmission was well correlated with an increase in the expression of the atrophy-associated gene, atrogin-1. This change may indicate muscle wasting rather than direct inflammatory process-associated damage. In the EAMG mice, there was a trend toward significant increase in local muscle inflammation as expressed by increased expression of MCP-1, IP-10, and CXCR3. This support a major role for peripheral immune response in the pathogenesis of the observed weakness in the EAMG mice but not in those injected with AQP4 peptide or antibodies. Additional possible explanation for the exacerbated muscle weakness and mild EAMG phenotype in the naive-injected mice could be due to the use of fatigability—MG-like examinations. We observed distinct fatigability and decreased grip force in both AQP4 peptide- and NMO-Ig-injected mice (Figure 2).

The exacerbated disease phenotype in EAMG mice injected with either AQP4 peptide or NMO-Ig may be explained by two pathogenic mechanisms: first, systemic and/or local immune response. EAMG mice as well as naive mice injected with NMO-Ig and EAMG with NMO-IgG revealed a significant increase in IL-6 secretion compared with CFA-injected mice. This is in accordance with the known IL-6 contribution to both NMO and MG [38–41] and the ample evidence for the role of the systemic immune response in MG and EAMG and disease severity [42]. Second, augmented activity of antigen-presenting cells. Both IL-10 and IL-6 are known to play a major role in NMO and MG [43, 44]. Increased secretion of IL-6 and lack of change in IL-10 were found in the cell cultures in our study supporting a divergence of the cytokine pattern and a possible role for antigen-presenting cells in mediating the pathological findings. The proinflammatory milieu can modify the immune balance; for example, increased IL-6 levels can alter T regulatory suppression activity and increase the resistance of effector T-cell suppression [42].

With regard to local immune response, the NMO autoantigen, AQP4, is also expressed in plasma membranes of cells outside the CNS, including skeletal muscle fibers. Several NMO patients have been reported with hyperCKemia. Recently, the histopathology of muscle involvement in NMO patients and hyperCKemia revealed mild endomysial and perivascular inflammation. Like the classic brain pathology found in patients with NMO, muscle lesions show lymphocyte and eosinophil infiltration with loss of immunoreactivity to AQP4 [16]. In our study, we found several indications that the AQP4 peptide- and NMO-Ig-injected mice had local muscle deviations. These included significantly decreased grip ability and signs of abnormal neuromuscular transmission with increased jitter and blocking on SFEMG. A possible mechanism by which NMO-Ig can exacerbate MG can be by interacting with the muscle AQP4, clustering more FC receptors and increasing the local neuromuscular junction pathology.

Additional possible scenario was raised by Guo et al., describing patients with NMO and hyperCKemia. The authors suggested that AQP4 may affect bioenergetic pathways and could be involved in the intracellular calcium dynamics of muscles [16]. Such mechanism can also explain the increased fatigability in EAMG and naive mice in our study. Calcium homeostasis is particularly perturbed in fast-twitch muscle fibers, which express AQP4 most abundantly and can therefore be affected by either NMO-Ig or by the specific immune response to AQP4 peptide. Furthermore, the report of mild myositis in EAE rats that received adoptively transferred AQP4-specific T-cells [45] is also in agreement to our findings.

Several animal studies have shown that the AQP4 antibodies are not pathogenic via simple transfer of AQP4 antibodies into naive animals. The NMO/EAE model clearly demonstrates that T-cells are required to facilitate the permeabilization of the BBB for the entry of antibodies and complement [21, 46–48]. We and the others have found increased AQP4-specific T-cell proliferation in patients with NMO [49, 50]. However, in the present study, T-cell proliferation was not affected by the stimulation with AQP4 peptide or NMO-Ig transfer.

Our approach of inducing a second autoimmune disease in mice that already developed one autoimmune syndrome can have clinical and pathophysiological relevance. Clinically, it is known that patients with one autoimmune disease tend to develop additional autoimmune disease [51, 52]. Immunologically, it is known that intermolecular epitope spreading occurs in animals and humans. For example, immunization of mice with peptides of the EBV, EBNA-1 protein, leads to the development of antibodies to SmB, SmD, nRNPs, and La/SSB [53]. Thus, immunization with the AQP4 peptide may increase immune reactivity to muscle epitopes, which can aggravate fatigability.

## 5. Conclusions

Our study shows exacerbation of disease severity in EAMG following immunization with the NMO autoantigen AQP4, or by NMO-Ig, that is accompanied by augmented inflammatory response. This finding could explain some of the clinical observations of increased susceptibility of patients with one autoimmune disease to develop a second autoimmune syndrome.

## Figures and Tables

**Table 1:** Aggravation of clinical severity and muscle strength in EAMG and naive mice by immunization with AQP4 peptide and NMO-IgG.

	EAMG control	EAMG + NMO-IgG	EAMG + AQP4 peptide	Naive + NMO-IgG	Naive + AQP4 peptide	Naive
Mean severity	0.624 ± 0.01	0.82 ± 0.01	1.5 ± 0.02^∗^	0.72 ± 0.01	0.63 ± 0.02	0
Gram force	242.8 ± 4.5	214.3 ± 4.5^∗^	219.4 ± 5.6^∗^	227.7 ± 8.9^∗∗^	219.6 ± 5.4^∗∗^	307.9 ± 7.4
Decrease in force (versus naive)	21.13%	30.40%	28.73%	26.03%	28.73%	0

∗ versus EAMG control. ∗∗ versus naive.

## Abbreviations

NMO: Neuromyelitis optica
EAMG: Experimental autoimmune myasthenia gravis
AQP4: Aquaporin 4
MG: Myasthenia gravis
AChR: Acetylcholine receptor
CNS: Central nervous system
IgG: Immunoglobulin G
EMG: Electromyography
SFEMG: Single fiber electromyography
BBB: Blood-brain barrier
EAE: Experimental autoimmune encephalitis
BgT: Bungarotoxin
SNEs: Sensory needle electrodes
CMAP: Compound muscle action potential
CN: Concentric electromyography needle
SFAPs: Single fiber action potentials
RNS: Repetitive nerve stimulation
TGF*β*: Transforming growth factor *β*
MCP-1: Monocyte chemoattractant protein
CXCR3: C-X-C motif chemokine receptor 3
IP-10: Interferon *γ* inducible protein 10.
